# Functional Hybrid Nanoemulsions for Sumatriptan Intranasal Delivery

**DOI:** 10.3389/fchem.2020.589503

**Published:** 2020-11-12

**Authors:** Lígia N. M. Ribeiro, Gustavo H. Rodrigues da Silva, Verônica M. Couto, Simone R. Castro, Márcia C. Breitkreitz, Carolina S. Martinez, Daniela E. Igartúa, Maria J. Prieto, Eneida de Paula

**Affiliations:** ^1^Department of Biochemistry and Tissue Biology, Institute of Biology, University of Campinas (UNICAMP), Campinas, Brazil; ^2^Department of Analytical Chemistry, Institute of Chemistry, University of Campinas, Campinas, Brazil; ^3^Department of Science and Technology, National University of Quilmes, Bernal, Argentina

**Keywords:** sumatriptan, hybrid nanoemulsions, vegetable oil, intranasal administration, biopolymers, nanotoxicity

## Abstract

In recent years, advanced nanohybrid materials processed as pharmaceuticals have proved to be very advantageous. Triptans, such as the commercially available intranasal sumatriptan (SMT), are drugs employed in the treatment of painful migraine symptoms. However, SMT effectiveness by the intranasal route is limited by its high hydrophilicity and poor mucoadhesion. Therefore, we designed hybrid nanoemulsions (NE) composed of copaiba oil as the organic component plus biopolymers (xanthan, pectin, alginate) solubilized in the continuous aqueous phase, aiming at the intranasal release of SMT (2% w/v). Firstly, drug-biopolymer complexes were optimized in order to decrease the hydrophilicity of SMT. The resultant complexes were further encapsulated in copaiba oil-based nanoparticles, forming NE formulations. Characterization by FTIR-ATR, DSC, and TEM techniques exposed details of the molecular arrangement of the hybrid systems. Long-term stability of the hybrid NE at 25°C was confirmed over a year, regarding size (~ 120 nm), polydispersity (~ 0.2), zeta potential (~ −25 mV), and nanoparticle concentration (~ 2.10^14^ particles/mL). SMT encapsulation efficiency in the formulations ranged between 41–69%, extending the *in vitro* release time of SMT from 5 h (free drug) to more than 24 h. The alginate-based NE was selected as the most desirable system and its *in vivo* nanotoxicity was evaluated in a zebrafish model. Hybrid NE treatment did not affect spontaneous movement or induce morphological changes in zebrafish larvae, and there was no evidence of mortality or cardiotoxicity after 48 h of treatment. With these results, we propose alginate-based nanoemulsions as a potential treatment for migraine pain.

## Introduction

Migraines are one of the top 10 causes of work disability in the world (Natoli et al., 2010; Vos et al., 2015). It is a painful and limiting disease with a prevalence above 20% (Yeh et al., 2018), characterized by periodic headache outbreaks, often associated with gastric problems and photo/phonophobia.

Although the disease mechanism is not fully understood, serotonin (5-hydroxytryptamine) is the probable trigger of migraine crises (Deen et al., 2019). In the late 1980s, some serotonin agonists, known as triptans, were developed for the treatment of migraine. Intranasal sumatriptan (SMT) is the gold standard treatment for severe migraines (Muzzi et al., 2020). However, SMT is a hydrophilic molecule, which limits its permeation through the nasal mucosa and makes it difficult to cross the blood-brain barrier (BBB), despite SMT action on the central nervous system (Pascual and Muñoz, 2005; Tfelt-Hansen, 2010). So far, there are no effective drugs available for treating migraines.

Nanoemulsions (NE) are drug delivery systems (DDS) in which at least 2 immiscible liquids are kinetically stabilized. They are mainly composed of aqueous and oily phases, with a huge loading capacity for hydrophobic molecules (Singh et al., 2017). They are able to prolong the drug release profile of several classes of drugs, improving their bioavailability (Rai et al., 2018). Moreover, NE with particle sizes smaller than 200 nm are especially promising to overcome the BBB. Indeed, NE have proved to be excellent DDS for the sustained release of drugs with neurological action (Islam et al., 2020; Nirale et al., 2020).

Additionally, NE composed of vegetable oils can take advantage of several therapeutic properties of these natural compounds (Badea et al., 2015; Ribeiro et al., 2017b). Copaiba oil is found in Central and Western Amazonia. It is composed of a mixture of triglycerides and fat acids (Ribeiro et al., 2017b), among which copalic acid is the major compound (Souza et al., 2020). The literature shows many reports on functional copaiba oil-based NE with remarkable anti-Leishmania (Dhorm Pimentel de Moraes et al., 2018), larvicidal (Rodrigues et al., 2014), anti-inflammatory (Lucca et al., 2018) and antimicrobial (Vaucher et al., 2015) activities. Moreover, the *in vivo* biocompatibility of copaiba oil was already reported in mice and rats (Alvarenga et al., 2020; Souza et al., 2020).

Biopolymers are natural materials that have been used in biomedical applications as DDS and biosensors, and in tissue engineering and diagnoses for at least 75 years (George et al., 2020; Qureshi et al., 2020). Due to their available carboxyl groups, hydrophilic biopolymers such as dextran, pectin (PCT), alginate (ALG), pullulan and xanthan (XAN) provide systems with mucoadhesive properties, favoring the permeation of drugs across the mucous tissue (Ribeiro et al., 2018b, 2020). Polymer-lipid DDS is one of the best combinations of pharmaceutical excipients, resulting in several optimized properties (Siepmann et al., 2019). In addition, biopolymers can be added to oily phases (Shinde et al., 2011) or complexed with hydrophilic molecules (Neupane et al., 2013), improving drug upload and *in vivo* efficacy, as noticed for other alginate-based lipid nanoformulations (Severino et al., 2015; Rodrigues da Silva et al., 2020).

In this work, we prepared different functional NE composed of copaiba oil and biopolymers complexed with SMT (2%) for intranasal administration. The long-term stability of NE was monitored for a year at room temperature. The most suitable formulation was structurally characterized and tested through *in vivo* nanotoxicity assays in a zebrafish model. The promising results evinced the potential of the hybrid alginate-nanoemulsion system for future clinical applications in the treatment of acute migraine crises.

## Materials and Methods

### Materials

Copaiba oil (*Copaifera langsdorfii*), Tween 20® (T-20), Tween 80® (T-80), xanthan gum from *Xanthomonas campestris* (XAN), alginate from brown algae (ALG), pectin from apple (PCT), and sumatriptan succinate (SMT) were purchased from Sigma-Aldrich (St Louis, MO, USA). Deionized water (18 mΩ) was obtained from an Elga ultrapure water purifier (Merck KGaA, Darmstadt, Germany). All other reagents were of analytical or pharmaceutical grade.

### Preparation of Biopolymer-SMT Complexes

Different biopolymer-SMT (1:4; w/w) complexes were obtained by homogenization of the excipients under magnetic stirring, at 25°C for 2 h. Then, such complexes were lyophilized, resulting in XAN-SMT, ALG-SMT, and PCT-SMT powder complexes, that were subsequently used as the active molecules in the hybrid nanoemulsion preparation.

### Preparation of Hybrid Nanoemulsions

Control NE were prepared as follows: the aqueous phase was obtained by adding 0.4 g T-80 plus, in which 0.05 g biopolymer (XAN, ALG or PCT) was solubilized in 10 mL of deionized water, under magnetic stirring (500 rpm), at 70°C for 10 min. The organic phase was simultaneously prepared, mixing 0.4 g T-20 and 1 g of copaiba oil, also under magnetic stirring at 70°C for 10 min. Then, the organic phase was dropped into the aqueous phase under constant agitation at the same temperature, for 10 min. The resultant copaiba oil-based emulsion was homogenized at 10,000 rpm for 3 min, using an Ultra-turrax machine (IKA® T18 basic). Finally, the microemulsion was ultrasonicated with a titanium micro-tip in a Vibracell machine (Sonics & Mat. Inc Danbury, USA) in cycles of 30 s (on/off) for 25 min (Dhorm Pimentel de Moraes et al., 2018). Finally, to prepare hybrid nanoemulsions with SMT (2%, w/v), the previously prepared, freeze-dried biopolymer-SMT complexes were added to the aqueous phase of NE, resulting in the NE/XAN-SMT, NE/ALG-SMT, and NE/PCT-SMT formulations.

### Long-Term Stability Study

The long-term stability of NE formulations with and without drug was monitored by following the parameters: particle size (nm), polydispersity index (PDI), Zeta potential (mV)—measured by Dynamic Light Scattering in a ZetaSizer Nano ZS 90 (Malvern Instruments, Malvern, Worcestershire, UK), and number of nanoparticles/mL—by Nanoparticle Tracking Analysis in a *NanoSight* NS300 instrument (Malvern Instruments, Malvern, Worcestershire, UK). The samples were diluted 1:2,000 and 1:250,000 for DLS and NTA measurements, respectively, and analyzed at predetermined time intervals for 1 year at 25°C (*n* = 3).

### Sumatriptan Encapsulation Efficiency (%EE)

SMT encapsulation efficiency (%EE) in the NE formulations was determined by the ultrafiltration-centrifugation method, followed by UV-vis quantification at λ = 227 nm (Agrawal et al., 2010). The concentration of SMT in the NE was determined by the difference between the non-encapsulated SMT quantified in the ultrafiltrate (free SMT) and the total amount of added sumatriptan (SMT initial concentration). The %EE was calculated according to the following equation:

(1) $$\begin{array}{r} \%\text{EE}=\text{A}/\text{B }x\;100 \end{array}$$

where: A is the amount of encapsulated SMT, and B is the SMT initial concentration; A = B – (free SMT).

### Sumatriptan *in vitro* Release Kinetics

The *in vitro* release of SMT (2%) was performed using Franz-type diffusion cells. The polycarbonate membrane was horizontally placed between the donor and acceptor compartments (area 1.77 cm^2^). The donor compartment was filled with 400 μL of each sample (free SMT, NE/ALG-SMT, and NE/PCT-SMT). The acceptor compartment (4 mL) was filled with 10 mM PBS solution to ensure the sink condition and kept at 37°C, under magnetic stirring (350 rpm). At predefined time intervals and up to 24 h, aliquots of 200 μL were removed from the acceptor compartment and immediately replaced with PBS. The concentration of SMT released during the experiment was photometrically determined at 227 nm (*n* = 5) and expressed as percent SMT released.

Modeling of the kinetic curves was carried out using KinetDS 3.0 software (Mendyk and Jachowicz, 2007). Among all the tested models (zero order, first order, Higuchi, Korsmeyer-Peppas, and Weibull), the Weibull model (Equation 2) showed the highest coefficient of determination (*R*^2^) values, for all the NE-based formulations:

(2) $$\begin{array}{r} m=1-exp\left[\frac{-{\left(t\right)}^{b}}{a}\right] \end{array}$$

where: *m* is the concentration of SMT released at the time *t, b* is the release exponent, and *a* is the time scale of release.

### Structural Characterization

FTIR-ATR, DSC, and TEM techniques were employed in the structural characterization of the excipients in nature, and the lyophilized ALG-SMT complex, NE, and NE-ALG/SMT samples.

FTIR-ATR spectra were collected by an infrared spectrometer equipped with ATR (Bruker IFS, Bruker, Billerica, MA, USA). The analyses were performed in transmittance mode, in the range of 4500–500 cm^−1^ with 2 cm^−1^ resolution. DSC thermal profiles were obtained with a TA Q20 calorimeter (TA Instruments, New Castle, DE, USA) equipped with a cooling system. Calibration was carried out using indium. The samples (5 mg) were added in aluminum pans and the spectra were detected from 0 to 200°C, at a 10°C/min heating rate, under nitrogen flow. The morphology of hybrid nanoemulsions, with and without drug, by TEM analyses was performed in a Leo 906 (Carl Zeiss, Oberkochen, Germany) transmission electron microscope, operating at 60 kV. Briefly, the samples were previously diluted (50 x) and a drop of the sample was added to the grid; after 1 min the excess was removed and a 2% uranyl solution (w/v) was added, providing contrast to the images. After 1 min the excess volume was removed, and a drop of deionized water was added to the sample that was dried at ambient temperature. Then, micrographs of the samples were obtained at different magnifications and the ImageJ software (US Nat. Institute of Health, Bethesda, USA) was employed to calculate the nanoparticle size from the micrographs.

### *In vivo* Zebrafish Tests

#### Maintenance and Spawning

Wild-type zebrafish (*Danio rerio*) were kept in tanks at a temperature of 28°C, with 14/10 light/dark cycles (Prieto et al., 2012). All embryos were collected by natural spawning and kept in Petri dishes with an E3 solution (5 mM NaCl, 0.17 mM KCl, 0.33 mM CaCl_2_, 0.33 mM MgSO_4_ at pH 7.0) (Kimmel et al., 1995). After 1-day post-fertilization (*dpf* ), they were transferred to a 96-well-plate with an E3 medium, three eggs per well, and maintained at 28 ± 0.5°C in a 14/10 h light/dark cycle, until 5 *dpf* . The procedures performed were approved by the ethics committees of the National University of Quilmes (CE-UNQ 2/2014, CICUAL-UNQ 013-15 e 014-15).

#### Viability and Determination of the Lethal Dose (LD_50_)

Five dpf larvae were exposed to SMT, NE/ALG-SMT, and NE/ALG, all diluted in an E3 medium. As a negative control, larvae were kept only in E3. The concentrations of SMT, free or encapsulated in NE/ALG-SMT, ranged from 2 × 10^−6^ to 2 × 10^4^ μg/mL. Controls prepared without SMT (NE/ALG) were used in the same particle concentrations of the NE/ALG-SMT formulation. For each assay, eight technical replicates and three biological replicates were used for each condition (*n* = 24). At 24 h post-incubation (hpi), larvae viability was observed in the stereomicroscope. Larvae were considered dead when they had no heartbeat and viability was expressed as the percentage of live larvae in relation to the control (larvae in the E3 medium). LD50 was determined as the drug concentration capable of causing the death of 50% of larvae (Martinez et al., 2019).

#### Heart Rate and Spontaneous Movement

For these tests, larvae were incubated at a sublethal concentration of 0.02 μg/mL SMT (SMT, NE/ALG-SMT) and NE/ALG for 24 h. Heart rate was determined by visually counting the larvae's heartbeats. The animals were immobilized on slides, placed under a stereomicroscope, and recorded in parasagittal orientation. The counting results were expressed as the percentage of beats per minute in relation to the control (larvae in the E3 medium). Spontaneous movement was analyzed in a multichannel ADC system (WMicrotracker, Designplus SRL) with infrared rays that are interrupted by the larvae's swimming activity (*n* = 24) (Igartúa et al., 2020). Spontaneous movement was expressed as the percentage of locomotor activity in relation to the control. Statistical analyses were performed with GraphPad Prism v.6.

#### Toxicity Analysis: Morphological Changes

For the study of morphological changes, after 24 *hpi* with the samples containing 2 × 10^−6^-2 × 10^−2^ μg/mL μg/mL SMT, the larvae were photographed in parasagittal orientation, with 60× magnification. The photomicrographs were analyzed for the following morphological changes: curvature of the body, malformation of the jaw, opacity of the head, opaque liver, opacity in the yolk sac, non-depletion of the yolk sac, uninflated swimming bladder, edema, and malformation of the tail. A score was assigned to each larva based on the degree of morphological anomalies: 0 = no anomalies; 1 = one to two morphological anomalies; 2 = three to four anomalies; 3 = more than four anomalies; and 4 = dead (Martinez et al., 2019). The average toxicity score for each treatment was determined by the score of the individual larvae.

### Statistical Analyses

One-way ANOVA followed by Tukey *post-hoc* multiple comparison tests were used to analyze significant differences over time of NE samples, in terms of nanoparticle size, PDI, Zeta potential and nanoparticle concentration, performed with R (version 4.0.1) analytical software. In addition, the same tests were employed to determine statistically significant differences between free sumatriptan and hybrid nanoemulsion, in the *in vivo* zebrafish model, regarding different parameters: viability, heart rate, spontaneous movement, and morphological changes. The significance level was defined as 5% (*p* < 0.05).

## Results

As a first approach, different complexes of biopolymer-SMT (1:4 w/w) were prepared in order to improve drug loading in copaiba oil-based nanoparticles. Then, copaiba oil-based NE formulations, containing or not the biopolymer-SMT complexes, were successfully prepared and stored for a year at room temperature.

### Long-Term Stability Study

**Figure 1:**
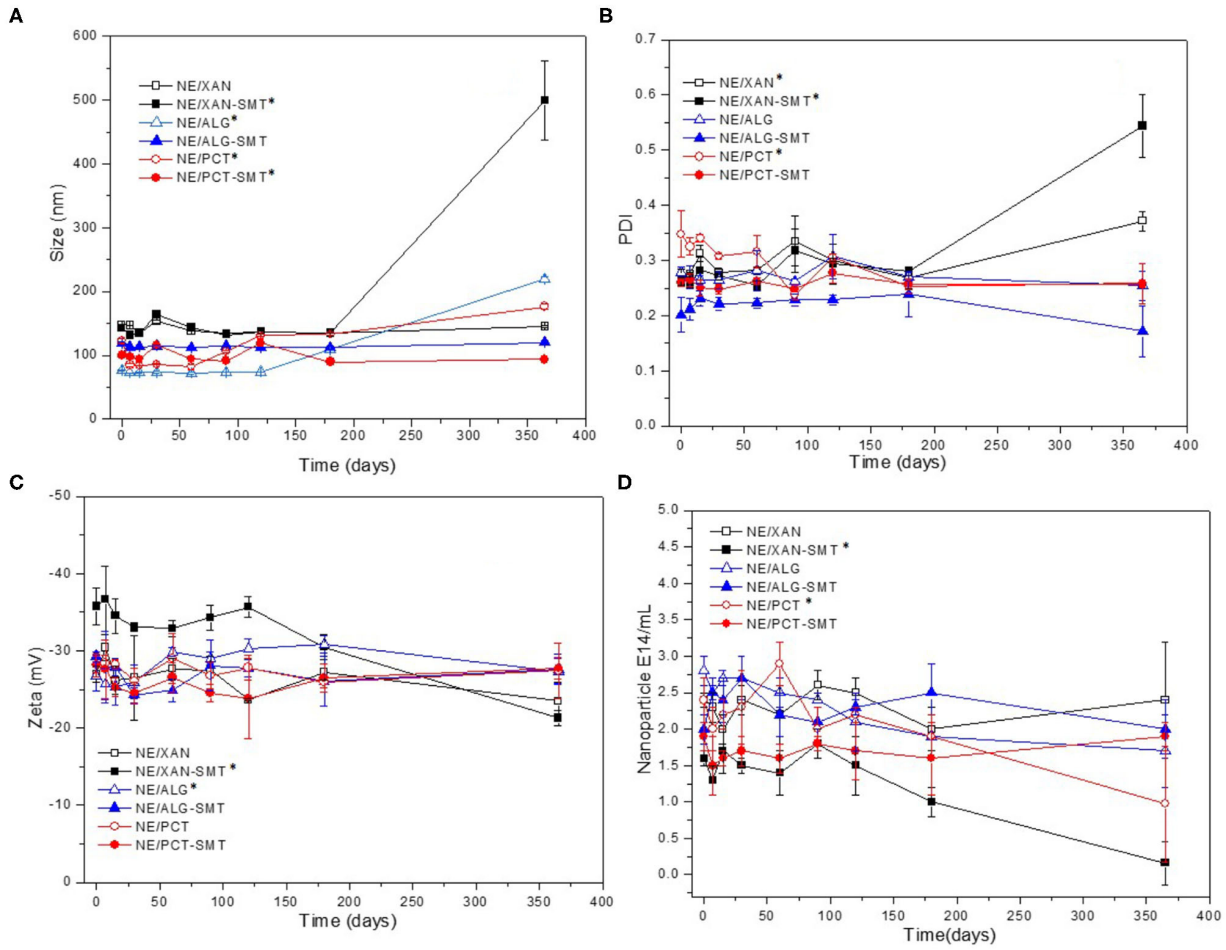
Long-term stability study of hybrid nanoemulsions—with or without SMT (2%)—in terms of size **(A)**, PDI **(B)**, Zeta potential **(C)**, and nanoparticle concentration **(D)**, during 365 days of storage at 25°C. Values are given as mean ± S.D. (*n* = 3) and ANOVA/Tukey *post-hoc* tests were used to differentiate intragroup statistically significant differences: *p < 0.05.

Hybrid NE formulations either containing or not containing SMT (2%) were monitored in the long-term stability study. The parameters analyzed for a year (25°C) were: size (nm), PDI, Zeta potential (mV), and nanoparticle concentration/mL (Figure 1). In general, initial particle size ranged from 76 to 148 nm (Figure 1A), with PDI values around 0.25 (Figure 1B), zeta potential from −26 to −35 mV (Figure 1C), and number of particles of 1.6–2.9 10^14^/mL (Figure 1D), for all NE.

NE/XAN-SMT exhibited statistically significant differences (*p* < 0.05) in all parameters tested after a year. NE/ALG-SMT was the only system that did not show any statistically significant difference (*p* > 0.05) after 365 days of storage, in any of the analyzed parameters. NE/PCT-SMT showed a significant increase in particle size (*p* < 0.05) at the end of the experiment. Taking into account such results, NE-XAN/SMT was excluded from subsequent (kinetic release and *in vivo*) experiments.

### Encapsulation Efficiency Test

SMT encapsulation efficiency (%EE) in the formulations is listed in Table 1. The hybrid NE formulations showed %EE values in the range of 41–69%, while NE/SMT encapsulated only *ca*. 10% of the drug.

**Table 1:** Sumatriptan (2%) encapsulation efficiency (%EE) in NE (without biopolymer in the composition) and in the hybrid biopolymer-NE formulations.

**Formulations**	**%EE**
NE/SMT	10.30 ± 4.52
NE/XAN-SMT	69.08 ± 7.92
NE/ALG-SMT	42.65 ± 1.04
NE/PCT-SMT	41.63 ± 12.20

*Values as mean ± S.D. (n = 3)*.

*XAN, 0.5% xanthan; ALG, 0.5 % alginate; PCT, 0.5% pectin*.

**Figure 2:**
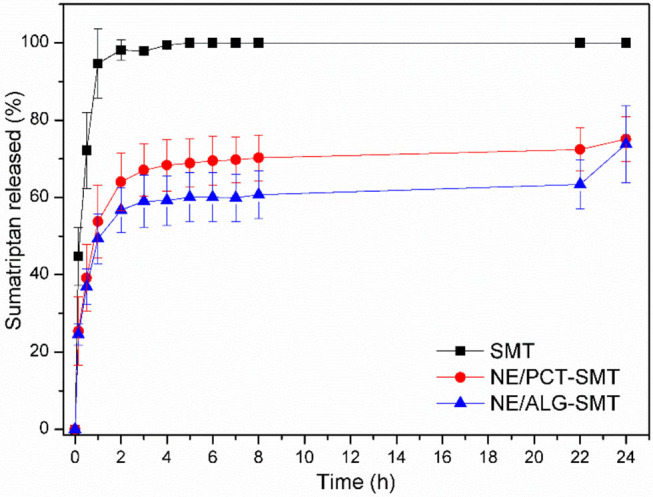
*In vitro* release kinetics of SMT, in solution or encapsulated in hybrid NE formulations, at 37°C, for 24 h. Data expressed as mean ± S.D. (*n* = 5).

Figure 2 shows the *in vitro* release profiles of SMT, free (control) or encapsulated in NE/ALG-SMT or NE/PCT-SMT. In the second hour of testing, SMT and NE samples discharged around 98 and 60%, respectively. After 5 h, SMT reached 100% of release in the acceptor compartment, while both hybrid NE released around 80% only after 24 h.

Mathematical modeling of NE kinetics was carried out using KinetDS 3.0 software. The best model was selected based on the highest coefficient of determination (*R*^2^). For SMT in solution, Higuchi was the best-fitted model, while the release kinetics of SMT in NE/ALG-SMT and NE/PCT-SMT was better described by the Weibull model (Table 2). The shape of the release curve (*b*-values) in the Weibull equation describes the mechanism of drug release. Here, the *b* < 1 values revealed a biphasic SMT release profile for both hybrid NE formulations (Papadopoulou et al., 2006).

**Table 2:** Mathematical modeling of release kinetic curves by the Weibull model.

**Nanoemulsion**	**Weibull**	
	***R***^**2**^	***b***
NE/PCT-SMT	0.92	0.22
NE/ALG-SMT	0.96	0.27

*R^2^, coefficient of determination; b, shape of release curve*.

Taking into account the long-term stability study, %EE and *in vitro* SMT release results, NE/ALG-SMT was selected as the most desirable formulation. Therefore, the structural characterization and *in vivo* nanotoxicity assays (zebrafish larvae model) were performed for NE/ALG-SMT, NE/ALG, and free SMT samples.

### Structural Characterization

The hybrid alginate-based NE containing or not SMT (NE/ALG-SMT and NE/ALG, respectively) were characterized using FTIR-ATR, DSC, and TEM techniques.

**Figure 3:**
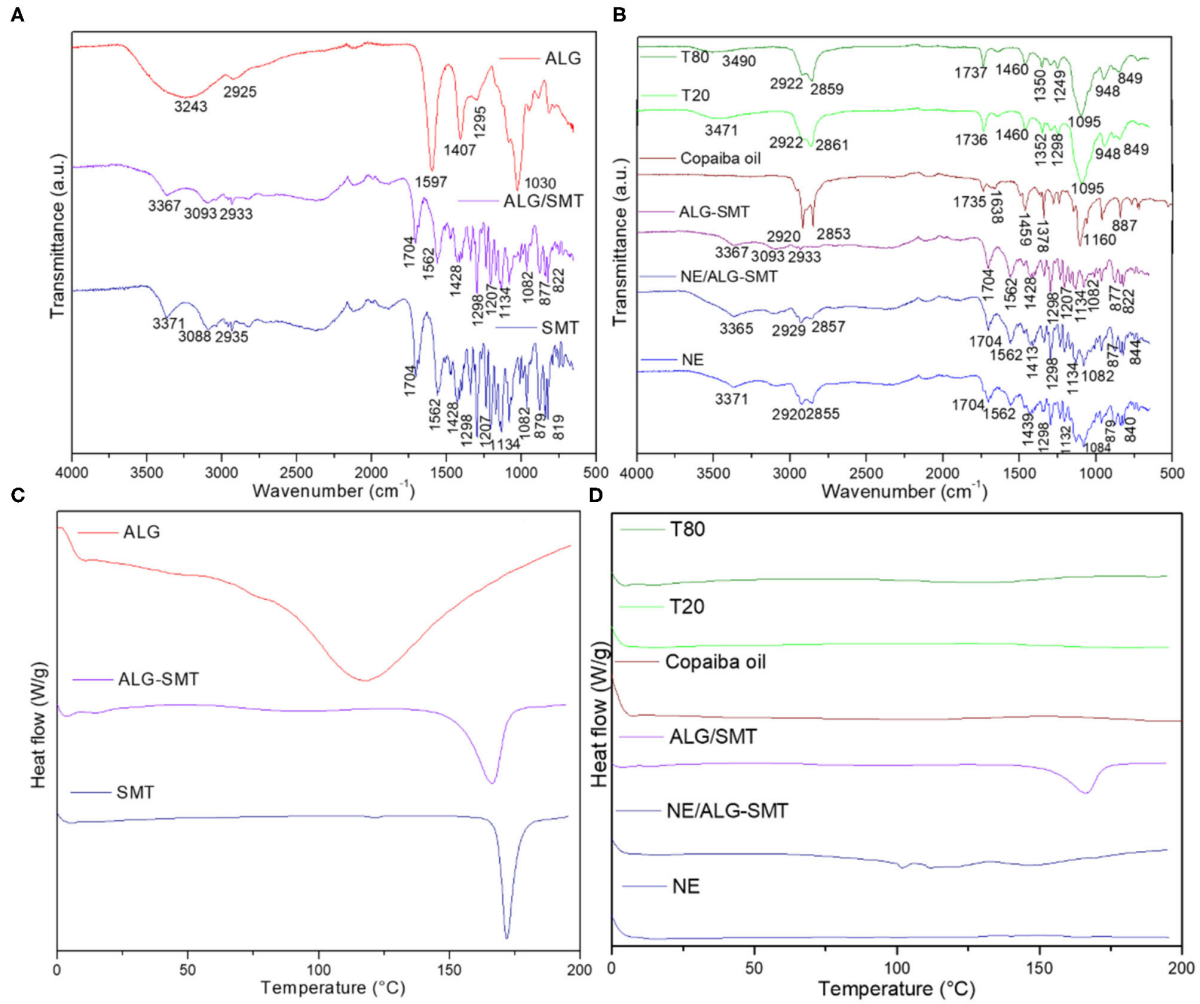
Characterization of the hybrid NE formulation and its excipients by the FTIR-ATR (top) and DSC (down) techniques. The structural profiles of the alginate-sumatriptan complex **(A,C)** and nanoemulsions **(B,D)** were provided.

Figure 3A shows the spectroscopic profile of the pure ALG, SMT, and ALG-SMT complex. Typical SMT bands were observed at 3371, 3088, 1298, 1207, and 1134 cm^−1^, ascribed to the stretching of -NH, C-H, sulfonamide, tertiary amine, and O-H (Galgatte et al., 2014), respectively, in the SMT spectrum. The spectrum of pure ALG displayed bands centered at 3243, 1587, 1407, and 1030 cm^−1^ assigned to O-H, -COO, -COO, and C-O-C stretching, respectively (Ribeiro et al., 2014). The ALG-SMT complex exhibited a spectroscopic profile similar to that of the SMT spectrum, where the SMT amine band (3371 cm^−1^) was shifted to 3367 cm^−1^. In addition, the broad band stretching band of -OH (3243 cm^−1^) from alginate was not detected in the complexed ALG-SMT spectrum.

Figure 3B features the FTIR-ATR spectra of the excipients, NE and NE/ALG-SMT formulations. NE (with and without SMT) spectra revealed bands in the regions of 2920–2929, 2855–2857, and 1132–1134 cm^−1^, corresponding to the stretching of CH, O-CH_2_, and C=O (Ribeiro et al., 2014), respectively. The spectrum of copaiba oil revealed bands centered at 2920, 2853, 1735, and 1160 cm ^−1^, corresponding to CH, O-CH_2_, C=O, and C=O stretching, respectively (Ribeiro et al., 2017b). In addition, the bands observed between 3365 and 3371 cm^−1^ in both NE and NE/ALG-SMT spectra are attributed to the interaction of copaiba oil with the hydroxyl groups of surfactants and biopolymer (Yu et al., 2012; Norcino et al., 2020).

Figure 3C features the thermodynamic transitions of the ALG-SMT complex, ALG, and SMT. It can be observed that the ALG-SMT complex showed an endothermic peak centered at 166°C, slightly lower than the melting point of pure SMT (172°C) (Galgatte et al., 2014) (Figure 3C). The melting point of the alginate (117°C) (Rodrigues da Silva et al., 2020) was not detected in the DSC analysis of the biopolymer-drug complex. Figure 3D shows the thermal profile of the excipients, NE, and NE/ALG, with or without SMT. All excipients (T-80, T-20, copaiba oil) were liquid at room temperature, and therefore did not show any thermodynamic transition in the analysis. Furthermore, there is no evidence of any peak of sample degradation up to 200°C.

**Figure 4:**
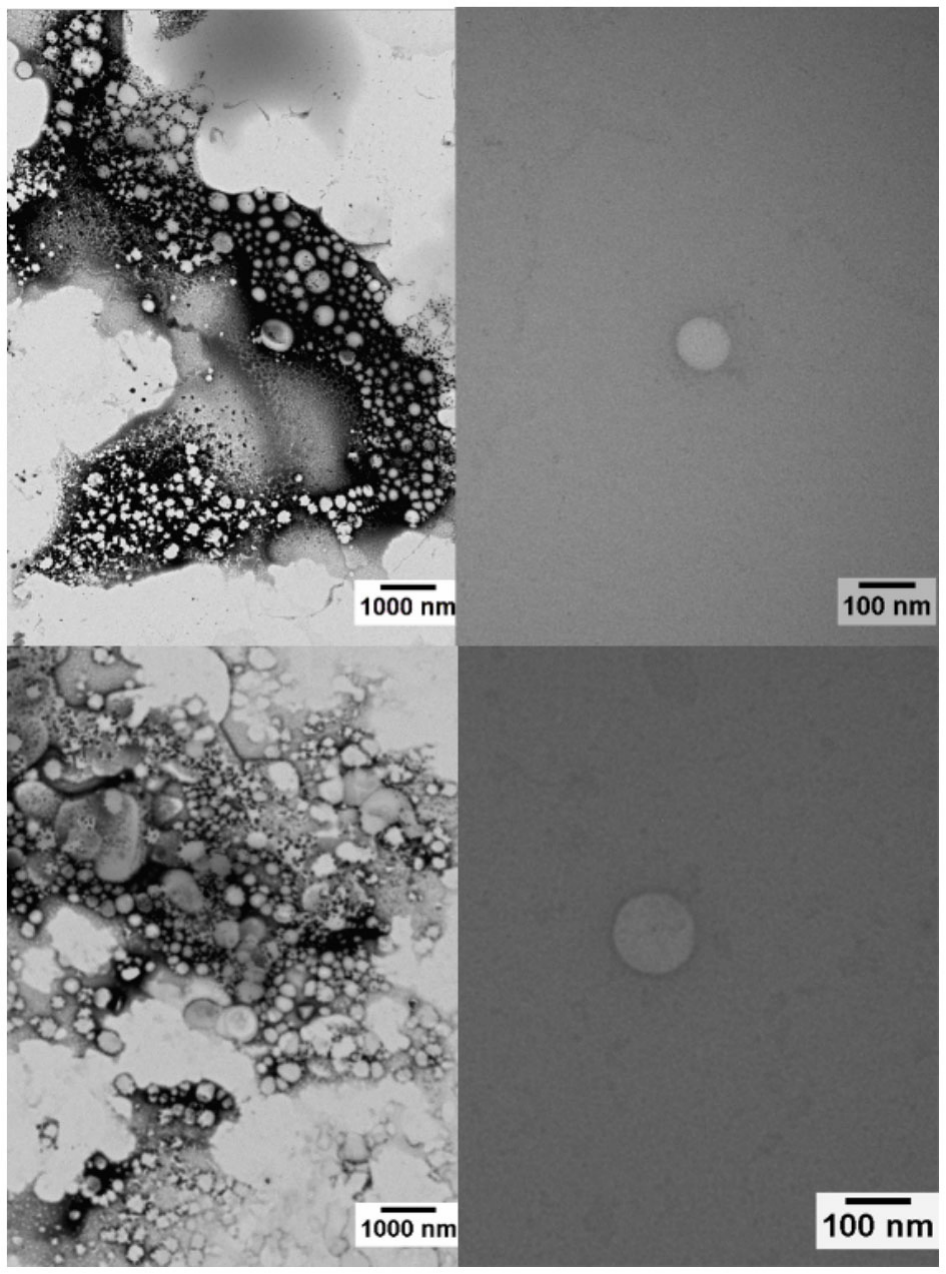
Micrographs of the NE/ALG (top) and NE/ALG-SMT (down) formulations, with magnifications at 10,000 x (left) and 100,000 x (right).

The micrographs of Figure 4 confirmed the spherical morphology of the nanoparticles and their narrow size distribution, as expected for this system (Kelmann et al., 2007). The incorporation of the ALG-SMT complex in NE did not interfere with the integrity and morphology of the nanoparticles. The particle sizes (around 94 and 123 nm for NE/ALG and NE/ALG-SMT, respectively) calculated from the micrographies using the ImageJ software were also in agreement with DLS data (Figure 1A).

### *In vivo* Zebrafish Tests

**Figure 5:**
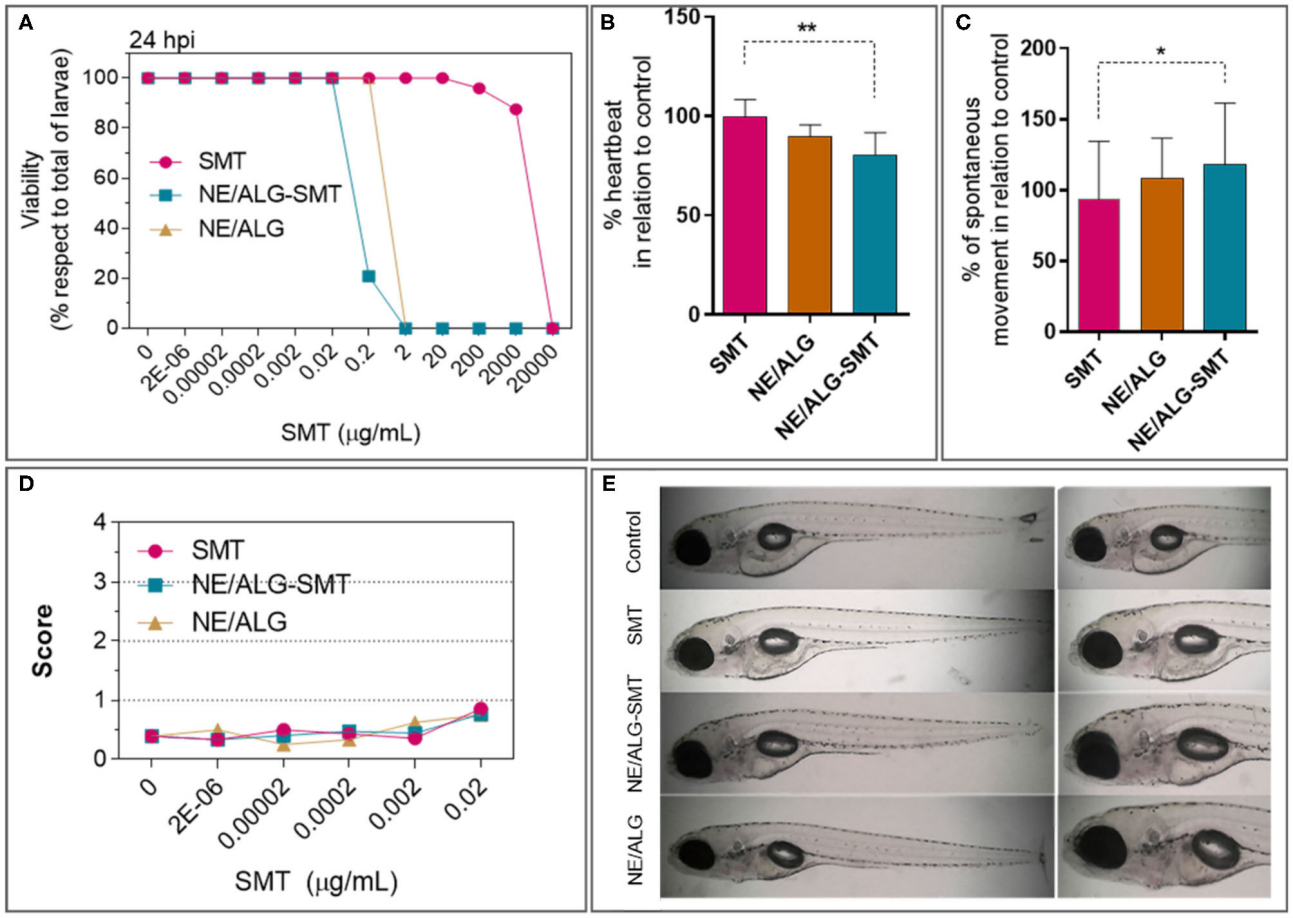
**(A)** Viability of zebrafish larvae after 24 h of exposure (*hpi*) to: sumatriptan in an aqueous solution (SMT), sumatriptan incorporated in the hybrid system (NE/ALG-SMT); control nanoemulsion without sumatriptan (NE/ALG). **(B)** Percentual variation of heart rate, in relation to control. **(C)** Percentual variation of spontaneous movement, compared to control. **(D)** Toxicological test: score of morphological changes after 24 *hpi*. **(E)** Representative images of the analyzed larvae after 24 *hpi*. Score: 0 = no changes; 1 = one to two morphological anomalies; 2 = three to four anomalies; 3 = more than four anomalies; and 4 = dead. Anova *post-hoc* Tukey test: *p < 0.05; **p < 0.01.

The first test performed on a zebrafish model was the determination of the dose capable of causing the death of 50% of larvae (LD_50_). The results found after 24 h post-incubation are given in Figure 5A. While free SMT showed LD_50_ = 3288 μg/mL, the NE-ALG/SMT formulation decreased this concentration to 0.2 μg/mL. A sublethal dose was then chosen in order to evaluate the action of the formulations on the target tissues (cardiac and neuronal). It is important to consider that the hybrid system without SMT (NE-ALG) also showed LD_50_ at a concentration equivalent to 0.6 μg/mL of SMT in the NE-ALG/SMT, or 3 × 10^−4^ mg/mL of copaiba oil or 6 × 10^8^ particles/mL.

Thus, heart rate (Figure 5B) and spontaneous movement (Figure 5C) were determined. Heart rate did not change in relation to the control when the larvae were incubated with free SMT. However, NE-ALG and NE-ALG/SMT decreased heart rate by 10 and 20%, respectively. Regarding the spontaneous movement of zebrafish larvae, a slight increase of 8% and >20% was observed for NE-ALG and NE-ALG/SMT, respectively, in relation to free SMT. In addition, to identify any possible systemic toxicity in larvae, several morphological parameters (as described in methods) were analyzed, and the mean score of each formulation is shown in Figure 5D, with representative images of the analyzed larvae in Figure 5E. The results show that in the tested concentrations, none of the formulations showed significant toxic effects, not reaching the score of 1. That is, <1 change was registered per larva, indicating systemic non-toxicity of the formulations.

## Discussion

The development of innovative nanostructured formulations aiming at the sustained release of active molecules requires satisfactory physicochemical properties, long-term stability, and biocompatibility (de Araújo et al., 2019; de Paula et al., 2020). Therefore, such features were pursued here.

It is essential to monitor some structural parameters of nanoparticles, such as particle size homogeneity and Zeta potential values to ensure quality control of pharmaceutical products (Attama et al., 2012). Nanoparticle disruption, coalescence, or degradation in colloids directly affect their potential as DDS. Thus, a novel *in vitro* biophysical method, called Nanoparticle Tracking Analysis (NTA), has been useful as an analytical method for nanoparticles (Filipe et al., 2010). In addition to the easy, fast, and reliable determination of particle size and polydispersity (Span index) of samples, this technique provides a unique piece of information, the number of nanoparticles in a known volume, without being affected by sample polydispersity or particle morphology (Ribeiro et al., 2018a).

NE-based XAN loading SMT was stable for only 6 months. After this period, average particle size (measured by DLS) increased significantly, followed by an abrupt decrease in the number of particles (measured by NTA). The strong negative correlation between these two parameters over time and measured by distinct techniques was already proposed by us as an instability indicator of colloids (Ribeiro et al., 2018a). NE/ALG-SMT was the only formulation that did not show significant changes in any of the analyzed parameters during long-term (365 days) storage. In addition, considering that the zeta potential is a parameter related with the non-colloidal stability, the highly negative values observed in here (−36 mV) confirmed the shelf-time of hybrid nanoemulsion (Honary and Zahir, 2013). In fact, ALG is the most successful biopolymer used as a carrier, absorption enhancer or adjuvant for DDS (Guo et al., 2020; Kuznetsova et al., 2020), as well as the polymer counterpart of organic-organic DDS (Ribeiro et al., 2017a; Siepmann et al., 2019).

The complexation of SMT with different biopolymers prior to NE preparation aimed to improve loading in copaiba oil nanoparticles. Such strategy has been previously described for modulating the hydrophilicity of other drugs encapsulated in lipid nanoparticles (Olbrich et al., 2004; Severino et al., 2015). In fact, all drug-biopolymer complexes were satisfactorily solubilized in the organic (nanoemulsion) phase, resulting in a DDS of higher SMT upload capacity than oily nanoparticles. Synergistically, such biopolymers impart an additional property to the system: mucoadhesion (Ribeiro et al., 2018b, 2020; Rodrigues da Silva et al., 2020), which is highly desirable for intranasal formulations.

Moreover, prolonged *in vitro* SMT release was observed for both NE/ALG-SMT and NE/PCT-SMT in comparison to free SMT. In addition, a burst release effect was observed for both NE in the first 2 h of analysis, which is desirable for pain management drugs (Franz-Montan et al., 2017a). The non-encapsulated fraction of SMT ensured the immediate onset of analgesia, followed by a sustained release of SMT loaded by copaiba oil nanoparticles. This biphasic release profile of NE is a consequence of its complex composition and unique supramolecular organization.

In this sense, the structural characterization of pharmaceutical products provided information of their molecular arrangement, as well as the possible interactions between the carriers and the drugs (Muniz et al., 2018). Here, FTIR-ATR, DSC, and TEM analyses evidenced the compatibility among all the components of the hybrid system. As expected, the ALG-SMT complex exhibited a similar spectroscopic profile to pure SMT, since there was 4 times more SMT than the biopolymer in the complex. In addition, the absence of the broad hydroxyl band from alginate and the shift of the SMT amine band to smaller wavenumbers in the ALG-SMT spectrum is indicative of electrostatic interactions taking place between the free amine of SMT and alginate carboxylic groups. Evidence of such interactions was also observed by DSC analyses, from the slight decrease of the SMT melting point in the ALG-SMT complex.

On the other hand, all NE spectra showed the typical spectroscopic profile of the lipid nanocarrier, reflecting the major contribution of copaiba oil to the NE structure (Ribeiro et al., 2017b). Thermal analyses also confirmed the compatibility of the excipients in NE/ALG-SMT. There was no evidence of a degradation peak in the NE/ALG-SMT thermal profile, suggesting interaction between the complex and NE with thermal stability to at least 200°C. Considering the physiological temperature range, around 35–37°C, these results indicated that NE can be applied as DDS without unexpected calorimetric transitions (Franz-Montan et al., 2017b).

The zebrafish model has emerged as an important preclinical tool to evaluate drugs and DDS (Berghmans et al., 2008; Fako and Furgeson, 2009; Haque and Ward, 2018; Jia et al., 2019). Practicality, low cost, quick testing of various parameters and good predictability with humans (>75%) are some of the factors that evidenced the importance of this model for nanotoxicity tests (Parng et al., 2002). Moreover, zebrafish larvae are transparent, which allows *in vivo* temporal imaging. In addition, the cardiovascular, nervous, and digestive systems are physiologically similar to mammals (Martinez et al., 2017). Specifically regarding the nervous system, it has been reported that the physiology of serotonergic neurotransmitters in zebrafish is similar to that of humans (Nowicki et al., 2014), with the development of BBB after 3 *dpf* (Eliceiri et al., 2011), thus being a coherent model for testing SMT (5HT1B and 5HT1D serotonin receptor agonist).

A particularity of the zebrafish model in relation to other models is the route of administration. In zebrafish, the larvae are exposed directly to the formulations in the bath solution. Thus, different pathways (gastrointestinal, dermal, etc.) can occur simultaneously in the larvae (van Pomeren et al., 2017). The viability results obtained revealed that high doses of free SMT are required to induce any effect on the larvae, as expected from its limited bioavailability. Conversely, NE/ALG-SMT showed the highest effect on larvae, which can be explained by the improvement of SMT bioavailability, as proposed in a recent review (Jia et al., 2019). This review showed that use of the zebrafish model to evaluate nanomaterials resulted in improved effectiveness of the encapsulated drug. Thus, the decrease in LD_50_ values of NE/ALG-SMT compared to free SMT is a strong indication of enhanced SMT delivery from NE/ALG-SMT. Moreover, it was no surprise that NE/ALG, composed of copaiba oil, had an effect on the larvae. Copaiba oil has exhibited several intrinsic therapeutic properties, such as analgesia (Gomes et al., 2007). However, when SMT is loaded in NE (despite only %EE 40%), the effect on viability is enhanced, indicating that encapsulated SMT had a higher impact on larval physiology.

In order to validate such a hypothesis, heart rate and spontaneous movement (reflecting the effects on the cardiac and nervous system, respectively) were analyzed. In both tests, SMT encapsulation potentiated the decrease of heart rate or the increase of spontaneous movement. These results confirmed the efficient delivery of SMT from NE/ALG-SMT, reaching these targets more efficiently. The changes in heart rate observed after treatment with free SMT should be correlated with its action in blood vessels (Caekebeke et al., 1992; Hack, 2004). In addition, copaiba oil was responsible for a decrease in larval heartbeat in NE/ALG by acting on opioid receptors (Leandro et al., 2012). Similar to the spontaneous movement results, the increase in larval movement when treated with encapsulated SMT should be a result of somatosensory discomfort, as already reported, e.g., discomfort to touch (Krämer et al., 2007).

Although these results indicated higher SMT bioavailability and synergy with copaiba oil effects in NE/ALG-SMT, no other systemic change was noticed in the tested doses, confirming the low toxicity of NE/ALG-SMT. Even though the zebrafish model does not reproduce the intended administration route (intranasal), it provided relevant insights into the bioavailability of SMT loading in NE, confirming the promising strategy to improve antimigraine triptan delivery.

## Conclusions

This work aimed at the development of hybrid nanoemulsions composed of copaiba oil and biopolymers for the loading of sumatriptan (2%) for intranasal administration. Firstly, the drug was complexed with different biopolymers to decrease its hydrophilicity and increase its upload by the copaiba oil-based nanoparticles. Then, those complexes were used as active molecules in NE. The NE/ALG-SMT formulation was stable for a year and prolonged *in vitro* SMT release for more than 24 h. Structural characterization revealed the singular supramolecular arrangement of NE, with electrostatic interactions detected between the biopolymer and SMT, plus hydrogen bonds between the complex and copaiba oil-nanoparticles. Finally, the *in vivo* nanotoxicity assays showed that NE/ALG-SMT was not toxic to zebrafish larvae in any of the analyzed parameters. Therefore, the nanoemulsion composed of copaiba oil plus alginate (0.5%) and sumatriptan (2%) is ready to be tested for *in vivo* efficacy in humans and subsequent clinical trials, aiming at migraine pain management.

## Data Availability Statement

The raw data supporting the conclusions of this article will be made available by the authors, without undue reservation.

## Ethics Statement

The animal study was reviewed and approved by Ethics committees of the National University of Quilmes, Argentina.

## Author Contributions

LR, VC, and EP proposed and designed all the experiments. LR, VC, SC, and GR conducted the preparation of formulation, stability, and release test. MB performed the structural characterization. MP, DI, and CM carried out the *in vivo* toxicity tests. LR, GR, EP, and MP wrote the manuscript. All authors contributed to the revision of the manuscript.

## Conflict of Interest

The authors declare that the research was conducted in the absence of any commercial or financial relationships that could be construed as a potential conflict of interest.
